# Undercounts of people with serious mental illness using the Washington Group Short Set questions

**DOI:** 10.3389/fpsyt.2025.1606154

**Published:** 2025-06-25

**Authors:** Jean P. Hall, Kathleen C. Thomas, Bryan P. McCormick, Noelle K. Kurth

**Affiliations:** ^1^ Institute for Health and Disability Policy Studies, University of Kansas, Lawrence, KS, United States; ^2^ Division of Pharmaceutical Outcomes and Policy, Eshelman School of Pharmacy, University of North Carolina at Chapel Hill, Chapel Hill, NC, United States; ^3^ Department of Health & Rehabilitation Sciences, Temple University, Philadelphia, PA, United States

**Keywords:** serious mental illness, disability, measurement, prevalence, WG-SS

## Abstract

**Background:**

Accurately counting Americans with mental health conditions is essential to support program development and appropriate resource allocations, which are often based on prevalence data. Multiple federal surveys use the Washington Group Short Set (WG-SS) questions to identify people with disabilities, including those with mental health conditions. However, the WG-SS questions miss many people with mental illnesses, under-representing this population in US federal survey data. Hence, we sought to explore the degree to which people with serious mental illness are missed.

**Methods:**

We used data from the 2020 National Survey on Health and Disability to assess the rates that respondents with self-reported serious mental illness (SMI) conditions, i.e., major depression, bipolar disorder, schizophrenia, and schizoaffective disorder (n=263), were missed as disabled by the WG-SS questions.

**Results:**

Using the three WG-SS questions suggested by the Washington Group to capture people with mental illnesses, 66.2%, 88.6%, and 96.6% of respondents with SMI were characterized as non-disabled; 58.2% were characterized as non-disabled across the three questions combined.

**Discussion:**

Previous research demonstrated that the WG-SS questions missed almost 60% of respondents with any mental illness. However, the Washington Group states that its question set better captures people with more severe disabilities, so this study focused only on respondents with serious mental illnesses and only on questions that the Washington Group suggests capture people with psychosocial disabilities.

**Conclusion:**

Results indicate that the WG-SS questions miss large percentages of even those with the most severe mental illnesses, who therefore may be substantially undercounted in US federal surveys using these questions. In turn, public mental health programs may be substantially underfunded.

## Introduction

Serious mental illness (SMI) is defined as “a mental, behavioral, or emotional disorder resulting in serious functional impairment, which substantially interferes with or limits one or more major life activities” and is estimated to affect approximately 5.5% of adults in the United States (1, 2). The SMI designation originated in the deinstitutionalization movement with a desire to replace terms such as “chronically mentally ill” or “persistent mental illness” with a term that was more indicative of possibilities for recovery (3, 4). Although several mental health conditions could be considered “serious,” the most prevalent diagnoses referenced in reviews of the SMI category have included schizophrenia, schizoaffective disorders, psychotic disorders, bipolar disorder, and major depression (5, 6).

In the US, the Americans with Disabilities Amendments Act defines a disability as “a physical or mental impairment that substantially limits one or more major life activities” and, further, a condition is considered a disability without regard to the ameliorative effects of mitigating measures such as medication, i.e., mitigating measures must be ignored in determining if an impairment substantially limits a major life activity (7). The law also states that an impairment that is episodic or in remission meets the definition of disability if it would substantially limit a major life activity when active. In fact, the appendix to the law specifically mentions major depressive disorder, bipolar disorder, and schizophrenia as examples of impairments that may be episodic but are still considered disabilities (8). Given these definitions above, people with SMI have a disability under US law.

Understanding the prevalence and characteristics of people with serious mental illness is essential for program planning and allocation of funds for services. For example, it is estimated that 26% of adults with SMI are covered by Medicaid (9) and people with SMI generate significantly greater Medicaid expenditures than adults without (10). The American Community Survey (ACS) is a national survey representative at the state and local government level that identifies rates of disability, including SMI, which are used to determine funding allocations and to support planning for federal, state, and local government services, including Medicaid and Medicare services. Additionally, ACS rates of disability are used to identify needs for mental health professional workforce development in compliance with a range of laws including the Social Security Act, the Americans with Disabilities Act, the Older Americans Act, and the Rehabilitation Act (11). ACS data are also used to understand critical gaps in the social, educational, and medical service systems, including for identifying populations at disproportionate risk of experiencing barriers to health care and poor health outcomes and for documenting and addressing discrimination in education and employment. In particular, cutting-edge strategies to understand and ameliorate the current mental health crisis are grounded in data that allow linkages of geographic, household, and individual characteristics within the policy context where people live, such as available through the ACS data (12).

Currently, the ACS uses a set of six yes/no questions, known as the “ACS-6,” to identify people with disabilities. Two of these questions specifically reference functional difficulties potentially due to a mental or emotional condition: *Because of a physical, mental, or emotional condition, do you have serious difficulty concentrating, remembering, or making decisions?* and *Because of a physical, mental, or emotional condition, do you have difficulty doing errands alone, such as visiting a doctor’s office or shopping?* While not exclusive to people with mental health conditions, these questions can be used to identify people with SMI (13). However, the Washington Group Short Set (WG-SS) (14) of disability questions was recently proposed to be used in the ACS, replacing the ACS-6 set (15), and is widely used for identifying people with disabilities in other US federal surveys (e.g., the National Health Interview Survey, the National Health and Nutrition Examination Survey, and the National Survey of Family Growth), despite the fact that federal testing indicated use of the WG-SS questions in the ACS would decrease overall disability estimates in the US by at least 40% (16). In contrast to the yes/no questions in the ACS-6, the WG-SS questions use a scaled response, asking how much difficulty respondents have with certain functions (no, some, a lot of difficulty, or cannot do at all; those answering “a lot of difficulty” or “cannot do at all” are counted as having a disability). One rationale for using the WG-SS questions in the ACS is that they are said to be better at identifying people with more severe disabilities. However, recent research, including our own, demonstrates that the WG-SS fails to capture people with severe hearing, vision, and mobility disabilities (17, 18). In fact, multiple studies have documented significant shortcomings of the WG-SS in identifying people with a variety of disabilities, including those with psychiatric disabilities (19, 20). The objective of this paper is to explore the degree to which the WG-SS questions accurately identified people with the most serious mental illnesses and to augment the literature regarding underperformance of the WG-SS questions in identifying people with the most severe disabilities.

## Materials and methods

### Data

We used data from the 2020 National Survey on Health and Disability (NSHD; n=2,175), a national online survey of American adults ages 18–64 with disabilities administered by researchers at the University of Kansas (21). All potential survey participants were screened with the question, “Do you have a physical or mental condition, impairment, or disability that affects your daily activities and/or that requires you to use special equipment or devices, such as a wheelchair, walker, TDD [telecommunications device for the deaf] or communication device?” Those who answered ‘yes’ to this question were invited to complete the survey and asked the WG-SS disability questions. All respondents were also asked to list all of their disabilities or health conditions in an open-ended response question. From the sample of respondents who answered all disability questions (n=2,099), we selected the 263 respondents who wrote in having major depressive disorder (n=107), bipolar disorder (n=129), or schizophrenia/schizoaffective disorder (n=37).

### Measures

While the WG-SS contains six questions, the Washington Group suggests (14) that people with psychosocial disabilities are identified primarily by their answers to the WG-SS questions about cognition (remembering and concentrating), communication, and self-care. Thus, we focused on responses to these three questions (see **Table 1** for question phrasing). Washington Group guidance indicates that those who answer “a lot of difficulty” or “cannot do at all” to these questions are considered disabled while people who answer only “some difficulty” or “no difficulty” do not count as disabled. As mentioned, SMI conditions are directly captured via self-report in the NSHD, allowing us to examine responses to these three WG-SS questions by people who reported having SMI. Other authors have documented the validity of self-report of these SMI conditions (22, 23).

**Table 1:** WG-SS psychosocial difficulties reported by NSHD respondents with serious mental illness (SMI)^a^ (n=263).

WG-SS item, level of difficulty	N	%
How much difficulty do you have remembering or concentrating?		
**No difficulty**	**27**	**10.3**
**Some difficulty**	**147**	**55.9**
A lot of difficulty	89	33.8
Can not do at all	0	–
How much difficulty do you have with self-care, such as washing all over or dressing?		
**No difficulty**	**154**	**58.6**
**Some difficulty**	**79**	**30.0**
A lot of difficulty	24	9.1
Can not do at all	6	2.3
Using your usual language, how much difficulty do you have communicating, for example understanding or being understood by others?		
**No difficulty**	**172**	**65.4**
**Some difficulty**	**82**	**31.2**
A lot of difficulty	9	3.4
Can not do at all	0	–
No difficulty or some difficulty to all three questions above		
	**153**	**58.2**

a SMI, individuals self-reporting major depressive disorder, bipolar disorder, schizophrenia or schizoaffective disorder to the question: “What is your disability and/or chronic health condition? If you have more than one, please list your main one first.” Bold indicates responses that would not be counted as having a disability.

## Results

Demographics for the NSHD group self-reporting SMI are shown in **Table 2**. In general, these demographics are similar to those for the overall NSHD sample of people self-reporting disabilities (also shown). As seen in **Table 1**, each of the three WG-SS questions said to capture people with mental health conditions missed more than half of respondents with SMI, because those who reported “no” or “some” difficulty with these functions would not have been identified as having a disability. Specifically, the question regarding difficulty with remembering or concentrating missed 66.2% of respondents with SMI; the question about difficulty with self-care missed 88.6% of respondents with SMI; and the question about difficulty with communication missed 96.6% of respondents with SMI. In combination, the three questions missed 58.2% of all respondents with SMI.

**Table 2:** Demographics of NSHD respondents with self-reported serious mental illness and those without self-reported serious mental illness.

Demographic	with SMI, % (n=263)	without SMI, % (n=1,836)
Age, mean years (SD, Range)	41.3 (13.1, 18-64)	42.0 (12.8, 18-64)
Gender		
Female Male Gender minority^a^ Did not report	60.5 30.8 5.3 3.4	64.5 33.7 2.7 0.1
Race and ethnicity		
White Non-white Did not report	72.2 21.3 6.5	80.1 17.8 2.1
Employment status		
Full-time Part-time Not employed	24.7 31.2 44.1	34.0 29.1 36.9
Household income^b^		
< 138% FPL 138-249% FPL 250-399% FPL > 400% FPL Did not report	44.5 22.1 13.3 16.3 3.8	35.1 22.2 19.3 22.3 1.0
Education level has college degree	56.1	60.5

a Gender minority includes: non-binary, transgender, genderqueer, genderfluid, gender non-conforming, and agender.

b FPL, Federal Poverty Level in 2019.

## Discussion

It is possible that not all people living with an SMI diagnosis would generally be considered disabled. Nevertheless, the Americans with Disabilities Act as amended specifies that a disability is any “physical or mental impairment that substantially limits one or more major life activities,” even when the impairments are mitigated by medications or accommodations. Individuals with SMI whose impairments are mitigated may need services and supports to maintain access to appropriate medications and accommodations. Availability and funding of such services and supports are facilitated through accurate prevalence estimates. Other research has recently demonstrated that the WG-SS questions underestimate disability prevalence in the US, including among those with the most severe vision, hearing, and mobility disabilities who may also benefit from services and accommodations (17, 18). Our findings suggest people with serious mental illnesses are also missed.

In a previous study using this dataset, we found that the WG-SS questions missed 58.7% of people reporting any mental illness, including those reporting potentially less severe conditions such as anxiety, personality disorders, or general mood disorders (24). For this study, we focused only on respondents with serious mental health conditions—those who self-reported experiencing major depressive disorder, bipolar disorder, schizophrenia or schizoaffective disorder—and found that the WG-SS questions still missed 58.2% of them. This finding suggests that the WG-SS questions may not more accurately capture people with more severe disabilities as stated by the Washington Group. More importantly, this finding has serious implications for our understanding of the true prevalence of people with these conditions and their experiences in accessing needed health services.

An important reason for the different outcome rates from the NSHD and WG-SS measures is that the WG-SS questions focus only on functional impairment across three domains whereas the NSHD uses a much broader screening question for disability, followed by prompts to write in all disabilities experienced. Functional impairment is not typically the basis of measurement of SMI (5). The current standard, established through the Mental Health Surveillance Study, is to combine functional limitation with assessment of psychological distress and its frequency and social limitations (25). Furthermore, a strength-based approach to assessment emphasizes positive gains, such as building a cohesive sense of self through personal agency, in addition to symptom reduction and functional improvement (26).

Based on these results, using the WG-SS has significant potential to undercount people with SMI. Such undercounting could result in contractions in service provision and eligibility, which can result in adverse outcomes. For example, Simpson et al. (27) concluded that Social Security policy contractions (e.g., reductions in eligibility) are linked to negative subsequent population mental health outcomes.

Limitations in this work are important to note. The NSHD is an internet-based survey, which may make it more accessible to respondents with greater resources and higher functioning. To the extent that people with greater limitations would answer in the affirmative to both the NSHD and WG-SS questions, the rate of under-reporting of the WG-SS may be lower than reported here. On the other hand, the NSHD may underreport people with psychiatric conditions who do not think of themselves as having a disability or psychological distress. Because about half of people with psychiatric conditions do not believe they have a condition that can be helped with healthcare services (28), they might not see this survey with its focus on health as relevant. Additionally, people with SMI in living arrangements supported by informal or formal/paid help may not experience functional disability or psychological distress to the extent of those with less support and thus not report them. Because people who do not see the NSHD as relevant to them would likely also answer that they do not have any of the 3 functional disabilities of the WG-SS question set, the true under-reporting of the WG-SS may be higher than reported here.

Future research should continue developing SMI self-report questions that are not deficit-based and allow for self-categorization of disability and/or open-ended responses to improve specificity of data (29). It has been 22 years since the last nationally representative psychiatric epidemiological survey was completed (30). We need a new one to make efficient investment of funds to understand current population needs and address unmet needs for people with SMI in the face of rising rates of mental distress (31). Precise measurement in existing national surveys is critical to motivate that investment.

Disability questions are used in a range of national surveys. It is vitally important to understand if, and to what extent, they capture people with SMI. This study indicates that the WG-SS disability questions miss many in this population and that more inclusive, and less deficit-based, questions are needed. The broad question used in the NSHD captured many people with self-reported SMI not captured by the WG-SS questions. Other similar broad disability questions are being tested and documented and this study reaffirms the need for their wider use in national surveys (18, 19). As Abdalla and Galea (32) note, the future of mental health epidemiology requires a refinement of definitions and methodologies, including in measurement.

## Data Availability

The raw data supporting the conclusions of this article will be made available by the authors, without undue reservation.
